# Steps Toward Minimal Reporting Standards for Lipidomics Mass Spectrometry in Biomedical Research Publications

**DOI:** 10.1161/CIRCGEN.120.003019

**Published:** 2020-11-16

**Authors:** Valerie B. O’Donnell, Garret A. FitzGerald, Robert C. Murphy, Gerhard Liebisch, Edward A. Dennis, Oswald Quehenberger, Shankar Subramaniam, Michael J.O. Wakelam

**Affiliations:** 1 Systems Immunity Research Institute, School of Medicine, Cardiff University, United Kingdom (V.B.O.).; 2 Institute for Translational Medicine and Therapeutics, Smilow Center for Translational Research, University of Pennsylvania, Philadelphia (G.A.F.).; 3 Department of Pharmacology, University of Colorado Denver, Aurora (R.C.M.).; 4 Institute of Clinical Chemistry and Laboratory Medicine, University of Regensburg, Germany (G.L.).; 5 Department of Chemistry and Biochemistry (E.A.D.), University of California, San Diego.; 6 Department of Pharmacology (O.Q.), University of California, San Diego.; 7 Department of Bioengineering (S.S.), University of California, San Diego.; 8 Babraham Institute, Babraham Research Campus, Cambridge, United Kingdom (M.J.O.W.).

**Keywords:** biomarkers, genomics, lipids, lipoproteins, publications

Lipids in blood and tissues can serve as markers of normal and pathophysiological function in humans and can even reflect functions in specific tissues and organs. Lipidomics describes the analysis of large numbers of lipids using mass spectrometry (MS). The proper implementation of these methods in a manner that ensures data quality requires care and rigorous manual checking. Issues of reproducibility and overall data quality in publications and guidelines for authors submitting research are well-developed for areas that include genetics/genomics, proteomics, and clinical trials. For example, the Human Proteome Organization has developed minimum information publication guidelines for proteomics (https://www.hupo.org/HUPO-Minimum-Information-Publication-Guidelines). However, apart from specialized lipid publications, such as the *Journal of Lipid Research*, which adopted the Lipid Metabolites and Pathways Strategy Consortium (LIPID MAPS) classification, nomenclature, and structural drawing formats in their guidelines,^1,2^ there are few reporting guidelines in use for lipidomics data. This issue is particularly relevant to studies that are not focused on underpinning methodological approaches but instead cover broader issues of human health and disease. In many such articles, multiple analytical methods are applied, making it difficult to engage sufficient technical expertise to afford rigorous and comprehensive review.

We developed a short set of guidelines for lipidomics submissions that we hope will contribute to improving reproducibility and standards in published work (Table). This is a living document, expected to be expanded as the field evolves. It is not intended to serve as a definitive final set of guidelines. To support this sort of activity, the Lipidomics Standard Initiative was recently established to create guidelines for major lipidomic workflows.^3^

**Table.**
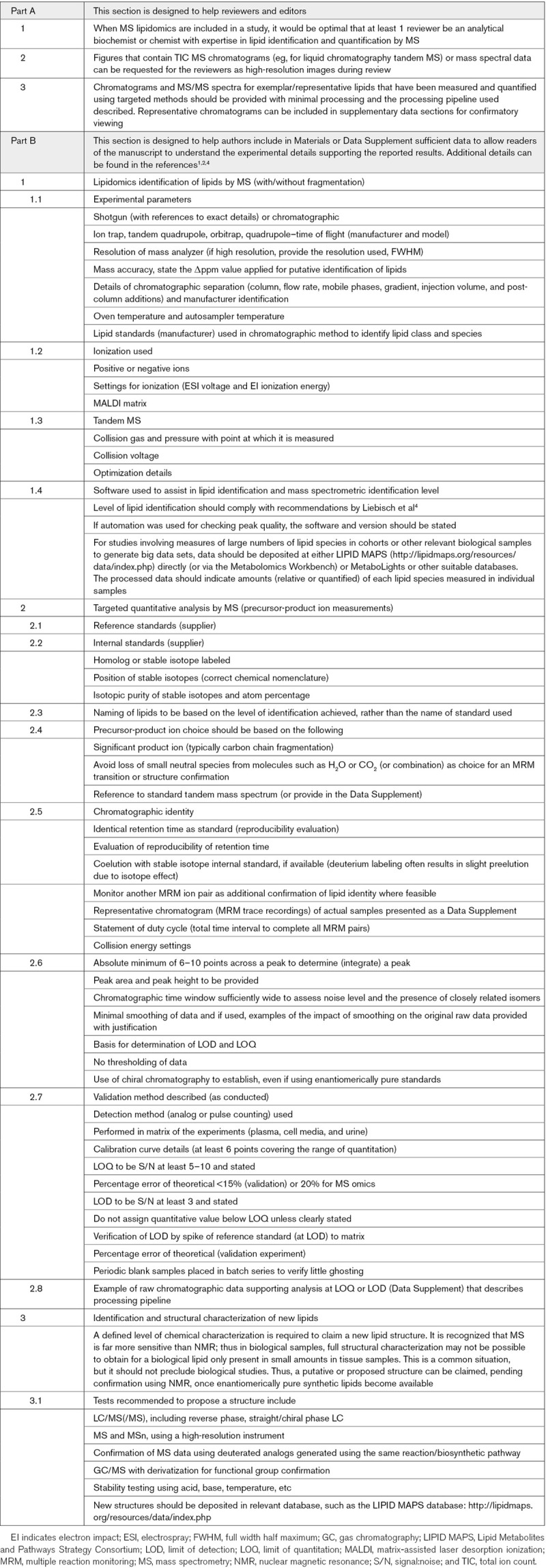
Guidelines

In the lipidomics field, different considerations apply to targeted and untargeted workflows, and it would be impossible to cover all of these in a short set of guidelines. However, we highlight some that we feel are worth special mention below.

## Guidelines For Manuscript Review

Given the complexity of this field, it is desirable that at least 1 individual with analytical domain expertise be included among the reviewers.

### Authentic Standards

Primary standards are generally highly defined in terms of stereochemistry, since they are chemically synthesized and thus highly purified single isomers or enantiomers. It is critical to confirm that the retention time of lipids in biological samples matches that for synthetic standards, as multiple isomers of lipids may overlap during elution. A separate issue relates to identification of complex lipids using MS without fragmentation, which does not define fatty-acyl composition. Here, appropriate shorthand annotation should be applied. Guidelines have already been developed concerning the type of MS used and the valid level of identification possible.^4^

### Nomenclature

One of the greatest sources of confusion in lipidomics research is the nomenclature of lipids. The wide use of disparate names for lipids and the lack of a standard naming system makes comparative analysis of lipidomics data across different studies generated by different equipment and investigators difficult. LIPID MAPS1,2 and the National Metabolomics Data Repository have tried to address this problem through the development of a Reference Set of Metabolite Names—a highly curated analytical chemistry-centric database of common names for lipids and other metabolite structures and isobaric species.^5^ Reference Set of Metabolite Names has been linked to the LIPID MAPS classification system enabling data sharing and meta-analysis. Easy tools for mapping and cross-referencing other lipid names have also been made available on the Metabolomics Workbench.

### Peak Quality

The fundamental analytical parameter is chromatographic resolution. Resolution may be enhanced by MS resolution of distinctive ion masses. Good peak shape is desirable and required when many analytes are recorded in a single run, but chromatographic and MS resolution is the criterion.

A pragmatic approach to assessing peak quality should be applied for targeted assays, especially since many laboratories are implementing new multiplex targeted methods that can quantify literally hundreds of lipids in a single run. A useful rule of thumb widely used in analytical chemistry would be that chromatographic peaks, generated from targeted multiple reaction monitoring experiments, which are usually gaussian in shape, should be at least 3× signal:noise for a limit of detection, rising to 5:1 or even 10:1 for defining the limit of quantitation. Care must be taken to be sure that any chromatogram used for analysis should have sufficient data points across the peaks analyzed (eg, preferably 6–10 data points across a peak) to identify its central retention time and peak shape enabling proper compound identification and quantification.

Determination of limit of detection and limit of quantitation, as well as the number of data points across a peak, should always be performed using raw data, with no smoothing applied. Representative raw data chromatograms (without any smoothing or other alterations) should be included for reviewers to inspect, and these may include expansions (blowups) of typical peaks used for signal:noise analysis and limit of detection/limit of quantitation determination. These should also be provided as published supplements or be deposited in suitable databases for readers.

Minimal smoothing may help to determine the apex of the peak and its shape relative to standards more accurately to assign the retention time, identify the compound, and quantify based on the area under the peak. Smoothing is primarily of use for cosmetically improving peaks, and since it enhances signal:noise, it should not be applied until data are tested for peak quality.^6^ A quick manual inspection of all raw data peaks will avoid computational errors that can be incurred if the automated determination of peak quality is solely applied using vendors’ software.

A separate use case is untargeted lipidomics, where lipids are detected based on high-resolution MS without fragmentation. Here, the identification of lipids is based only on accurate mass and retention time. Including spectral data in the acquisition process provides additional information for structural identification and validation, such as fatty acid composition of complex lipids. Here, automated methods are becoming more generally applied, and their use is likely to increase in the future. In the attempt to automate the identification, integration, and interpretation of MS data, peak analysis becomes critical. But with software-guided analysis, there is often the need to make some compromises to include all peaks of interest in the specific analysis. However, it is always important to at least spot-check peaks across the resulting chromatogram to verify the software-generated assignment of the relevant lipids of interest to the investigator, as well as the peak (and its shape) used for integration. One useful tool that may help with this is Lipid Data Analyzer (http://genome.tugraz.at/lda2/lda_download.shtml).^7^ This can be downloaded free of charge or used online. The original version is optimized for the annotation of phospholipids and glycerolipids and is platform independent.

### Quantitation of Lipids

Methods to carry out quantitative analysis by MS include stable isotope dilution and targeting specific lipids. This is the approach typically used for those lipids present at low abundance and for which reference standard material and isotope-labeled internal standards are available. Alternative methods for those lipids present as multiple molecular species utilize an unnatural species (homolog or isotope labeled) as an internal standard and a specific reference lipid to generate several standard curves. The mass spectrometer can be operated in a nontargeted or targeted (tandem precursor/product monitoring) mode, and each approach has advantages and disadvantages. Rather than absolute quantitation, relative quantitation can be used in a controlled experimental series that does not require standard curve generation and is accurate for fold changes. The use of the same m/z value for precursor-to-product ion (multiple reaction monitoring) experiments is discouraged since it does not allow lipids with the same m/z to be discriminated if they coelute; thus for quantitative methods, a product ion that is unique to the lipid of interest should be chosen, if possible. If the same m/z value is required, then in such cases, chromatography should provide sufficient specificity.

### Structural Analysis of Lipids

We suggest a pragmatic approach to the description of new lipid mediators, reporting putative structures based on the information available. As an example, with a newly discovered lipid, part of the structure (eg, carbon chain length, number of rings/double bonds, position of oxygenation, and nature of functional groups) may be known, but details that include stereochemistry or double bond isomers may not yet be elucidated. Here, the known biological information such as enzymatic and cellular source and putative bioactivity can be placed in the public domain to encourage others to follow the work and expand it, including through more detailed structural characterization. Supporting publication of structures where the full stereochemistry may not yet be known ensures that ongoing biological studies can proceed but has the advantage of enabling updates to be provided as additional information, such as nuclear magnetic resonance and chiral identification become available. Where reference spectra are included, the major product ions should match the synthetic standard.

### Data Deposition

In relation to untargeted workflows, large datasets should be deposited at recognized repositories for future data mining and integration into systems biology, such as the Metabolomics Workbench (https://www.metabolomicsworkbench.org/repository/index.php), which has a portal through LIPID MAPS (http://lipidmaps.org/resources/data/index.php), or MetaboLights (https://www.ebi.ac.uk/metabolights/presubmit). We recommend the LIPID MAPS nomenclature and Reference Set of Metabolite Names be used as a common standard approach, either the shorthand or fully annotated nomenclature as appropriate.^1,2^

Last, we highlight that there are established international guidelines for validation of analytical procedures, from the World Health Organization, Food and Drug Administration, and European Medicines Agency. These are designed for drug or toxicology applications that require a higher level of validation than research assays in general, but they provide excellent information relating to accepted approaches for validation of quantitative methods in the field, as follows: https://www.who.int/medicines/areas/quality_safety/quality_assurance/28092018Guideline_Validation_AnalyticalMethodValidation-Appendix4_QAS16-671.pdf; https://www.fda.gov/regulatory-information/search-fda-guidance-documents/analytical-procedures-and-methods-validation-drugs-and-biologics; https://www.ema.europa.eu/en/ich-q2-r1-validation-analytical-procedures-text-methodology.

## Sources of Funding

Funding from the Wellcome Trust for LIPID MAPS is gratefully acknowledged (203014/Z/16/Z). LIPID MAPS receives sponsorship from Avanti Polar Lipids, Cayman Chemical, and Merck. This study was supported by National Institutes of Health grants UL1 TR001878 (Dr FitzGerald), GM20501 (Dr Dennis), U54 GM069338 (Drs Dennis, Murphy, Subramaniam, and Quehenberger), U01 DK097430 Metabolomics Workbench (Dr Subramaniam), and U2C DK119886 Metabolomics Workbench (Dr Subramaniam). Dr O’Donnell is a Royal Society Wolfson Merit Awardee.

## Disclosures

None.
